# Estimation of the Effectiveness of a Tighter, Reinforced Quarantine for the Coronavirus Disease 2019 (COVID-19) Outbreak: Analysis of the Third Wave in South Korea

**DOI:** 10.3390/jpm13030402

**Published:** 2023-02-24

**Authors:** Marn Joon Park, Ji Ho Choi, Jae Hoon Cho

**Affiliations:** 1 Department of Otorhinolaryngology-Head and Neck Surgery, Inha University Hospital, Inha University School of Medicine, 27, Inhang-ro, Jung-gu, Incheon 22332, Republic of Korea; 2 Department of Otorhinolaryngology-Head and Neck Surgery, Soonchunhyang University College of Medicine, Bucheon Hospital, 170, Jomaru-ro, Bucheon 14584, Republic of Korea; 3 Department of Otorhinolaryngology-Head and Neck Surgery, Konkuk University School of Medicine, 120-1, Neungdong-ro, Gwangjin-gu, Seoul 05030, Republic of Korea

**Keywords:** COVID-19, coronavirus disease 2019, SARS-CoV-2, quarantine, Republic of Korea, South Korea, ARIMA

## Abstract

It has been claimed that a tighter, reinforced quarantine strategy was advocated to reduce the transmission of coronavirus disease 2019 (COVID-19) during major outbreaks; however, there have been no prior quantitative studies examining the effectiveness and duration of such a reinforced quarantine. Consequently, the purpose of this research was to determine the impact of a “tighter, reinforced” quarantine during the third COVID-19 breakout wave in South Korea, which occurred between late 2020 and early 2021. The efficacy of the quarantine was determined by comparing the number of newly diagnosed COVID-19 patients between the “prediction model” and “actual observed data.” Two prediction models were developed using the autoregressive integrated moving average (ARIMA; 1, 0, 0) model. The effect of a “tighter, reinforced” quarantine, which would show as an immediate drop in the number of new cases, predicted its efficacy by lowering the number of new cases by 20,400. In addition, the efficacy of the quarantine lasted up to more than three months. The findings of our investigation confirmed the beneficial influence of “tighter, controlled” quarantine laws during a widespread COVID-19 epidemic. During an epidemic, when the population has not yet developed immunity to respiratory viral diseases, our study may be evidence for implementing stricter quarantine restrictions in order to reduce the number of new cases.

## 1. Introduction

The severe acute respiratory syndrome coronavirus 2 (SARS-CoV-2), which was formerly known as 2019-nCoV [1], is the infectious agent that causes the human illness known as coronavirus disease 2019 (COVID-19), which primarily affects the respiratory system in humans [2]. The COVID-19 virus was discovered for the first time in December 2019 in Wuhan, China, and since then, it has rapidly disseminated around the world, resulting in a pandemic that has affected the whole world [1].

Despite the fact that more than half of patients (81%) report only having moderate disease severity, COVID-19 has the potential to cause severe sickness (14%) and critical illness (5%), as well as having a mortality risk of 2.3% [3]. According to research that was released in 2020, COVID-19 is mostly transmitted by respiratory particles and has a basic reproduction number (R0) ranging from 1.4 to 2.4 [4]. In light of the fact that asymptomatic COVID-19 carriers are also likely to transfer the disease to others [5,6], an R0 of more than 1 in COVID-19 suggests that this disease will spread if a community-wide intervention is not put into place to reduce the amount of direct person-to-person contact [4]. As a consequence of this phenomenon, it cannot be emphasized enough how important it is to quickly detect, confirm, and manage the ill individual, in addition to implementing stringent national quarantine standards and a healthcare system that is overseen by each government [7].

In the year 2020, the COVID-19 epidemic spread over South Korea in three separate “waves” [8]. In response to the severity of the COVID-19 epidemic, the government of South Korea implemented a degree of quarantine that severely restricted public activities and social gatherings in the country [9]. In spite of the measures that were taken, the number of individuals who were newly diagnosed with COVID-19 grew considerably between November 2020 and February 2021 [8]. In contrast with the first and second waves, the third wave of COVID-19 reached a higher number of newly diagnosed patients each day than the first and second waves combined, and it demonstrated a random pattern over the whole of the nation [10]. As a result, the authorities of South Korea felt they had no choice but to implement “increased” quarantine and social gathering restrictions [10].

As a result of the “increased” legal standards, there were fewer patients newly diagnosed with COVID-19 [10]. Nevertheless, it is essential to comprehend that the inevitable expansion of lockdowns and limits on social activities put a major pressure on the South Korean economy and triggered a financial emergency for a great number of people living in the community [11]. Therefore, it is still a million-dollar question whether or not the “tighter, enhanced quarantine” will be helpful in the future in preventing the spread of COVID-19 during certain outbreaks.

Although a great deal of research on COVID-19 has been published, very few studies have investigated the “efficacy” of strengthened quarantine measures in preventing the spread of COVID-19 at either the community or the national level. This is in spite of the fact that there has been a great deal of research on COVID-19 published. In addition, a number of studies that were carried out in the past have shown that the legislation governing quarantine had a good impact on the quality of quarantine effectiveness [12]. On the other hand, there has not been a single piece of research that has made an effort to quantify the impacts of quarantine reinforcement. As a consequence of this, the goal of this study was to develop a forecast and assess the efficacy of “enhanced quarantine” measures during the third wave of COVID-19 outbreaks in South Korea in the year 2020.

## 2. Materials and Methods

This research was conducted in accordance with the ethical principles outlined in the Declaration of Helsinki from 1975, and the current study received approval from the Institutional Review Board (IRB) of the Inha University Hospital (IRB No.: 2022-12-021).

During the third wave of the COVID-19 outbreak that spread throughout South Korea, the major objective of the current research was to analyze the effectiveness of the strengthened quarantine restrictions. Calculations were performed to determine the degree to which the “prediction model” and the “actual data” differed in the number of COVID-19 patients who were newly diagnosed with the disease. This was undertaken so that the impact of stricter quarantine regulations could be evaluated.

In the past, a number of different models—including compartmental models, agent-based models, and time-series models—were used to predict and forecast the number of people who were infected with a range of different transmissible illnesses [12,13]. We decided to use the non-seasonal autoregressive integrated moving average (ARIMA) model to forecast the estimated number of newly diagnosed COVID-19 patients in South Korea based on the analysis of the auto-correlation and partial auto-correlation coefficients in the raw data of daily diagnosed COVID-19 patients. This decision was reached as a result of the analysis of the auto-correlation and partial auto-correlation coefficients in the raw data of daily diagnosed COVID-19 patients.

In essence, the autoregressive integrated moving average (ARIMA) model is a mathematical tool that makes projections about time-dependent serial values in the future. These projections are derived from data that are based on previous sequences of time-dependent serial values. We decided to use a non-seasonal ARIMA (1, 0, 0) model, which is also known as a “first-order autoregressive model.” This model was chosen from among the non-seasonal ARIMA models that have been categorized. The ARIMA (1, 0, 0) model is a prediction model that is based on the assumption that if a certain series of values are stationary and autocorrelated, then this model may possibly enable the prediction of a series of values in the future, calculated from the past data that were entered. This model works on the assumption that a certain series of values are stationary and autocorrelated. All statistical analysis was performed using IBM SPSS Statistics for Windows, Armonk, NY, USA: IBM Corp.

We developed two models in order to make an accurate prediction of the number of newly diagnosed COVID-19 patients. These models took into account two distinct time periods and calculated the projected number of newly diagnosed COVID-19 cases from that information. The first model was constructed from data from a time period prior to the implementation of “tighter, reinforced quarantine.” We gathered the reported data on newly diagnosed COVID-19 patients in South Korea, which were released by the Ministry of Health and Welfare of the South Korean government [14]. According to the findings of our research, the period of time before the implementation of the stricter and more stringent quarantine was determined as occurring between 21 September 2019 and 24 December 2020. The second model was built using data from the age of “tighter, reinforced quarantine,” which was defined as the time period from 25 December 2020 to 13 April 2021. In each time period, data on the daily number of people who received a new diagnosis of COVID-19 were gathered, which allowed for the creation of two distinct models.

Both models, using the datasets that were entered into them, made projections about the number of newly developing instances of COVID-19 in the future. A bar-line graph was generated by using the observed actual number of new cases in conjunction with the projected number of fresh-onset COVID-19 cases. A comparison was made between the number of cases anticipated model, and the actual number that was observed, and the time period that demonstrated a difference between the two models was studied.

**Figure 1:**
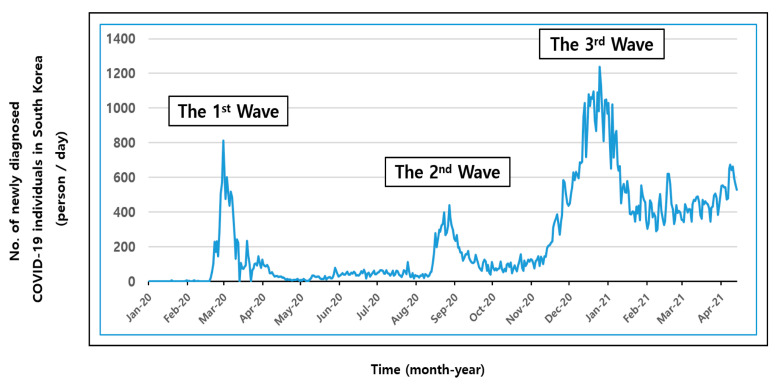
The number of newly diagnosed coronavirus disease 2019 (COVID-19) patients in South Korea (January 2020 to April 2021) shown in a chronological sequence. The number of newly diagnosed COVID-19 patients peaked in January 2020, February 2020, and April 2021. The 3rd wave of South Korea’s COVID-19 epidemic began in mid-December 2020 when 600 cases were reported and 1200 were confirmed by 25 December. From mid-January 2021, the number of newly confirmed cases reduced due to South Korea’s quarantine policies.

Since the first instance of COVID-19 was verified in South Korea on 19 January 2020, there were three outbreaks between 2020 and 2021, which was an era prior to the distribution of a COVID-19 vaccine to the public [8] (Figure 1). As illustrated in Figure 1, three peak waves (1st, 2nd, and 3rd) on the number of newly diagnosed COVID-19 individuals from January 2020 to April 2021 are shown. The number of confirmed patients explosively increased during mid-December in 2020, surpassing 600 on 4 December and 1200 on 25 December, and this outbreak was known as the 3rd wave of the COVID-19 outbreak in South Korea. With the reinforcement in the quarantine policy by the South Korean government, the number of newly diagnosed cases decreased from around mid-January 2021.

The beginning of the third wave had an official start date of 22 October 2020, which was the day when the number of new COVID-19 infections reached epidemic proportions (Figure 1). Although the quarantine was announced on 24 December 2020 [14], given that the typical incubation time of COVID-19 was about one week [15], the full impact of “tighter, reinforced quarantine” officially began on 31 December 2020. Therefore, the time before the “tighter, reinforced quarantine” was described as occurring between 22 October 2020 and 30 December 2020, and the time during the “tighter, reinforced quarantine era” was classified as occurring between 31 December 2020 and 13 April 2021. This was arranged so that the “tighter, reinforced quarantine era” would begin on 31 December 2020 and end on 13 April 2021.

## 3. Results

As depicted in Figure 1, the highest number of newly diagnosed COVID-19 patients was seen in January 2020, followed by February 2020, and then April 2021. The third wave of the COVID-19 outbreak that was sweeping South Korea started in the middle of December 2020, when 600 cases were detected. By the 25th of that month, 1200 cases had been confirmed. Due to South Korea’s quarantine regulations, there was a decrease in the number of newly confirmed cases beginning in the middle of January 2021.

**Figure 2:**
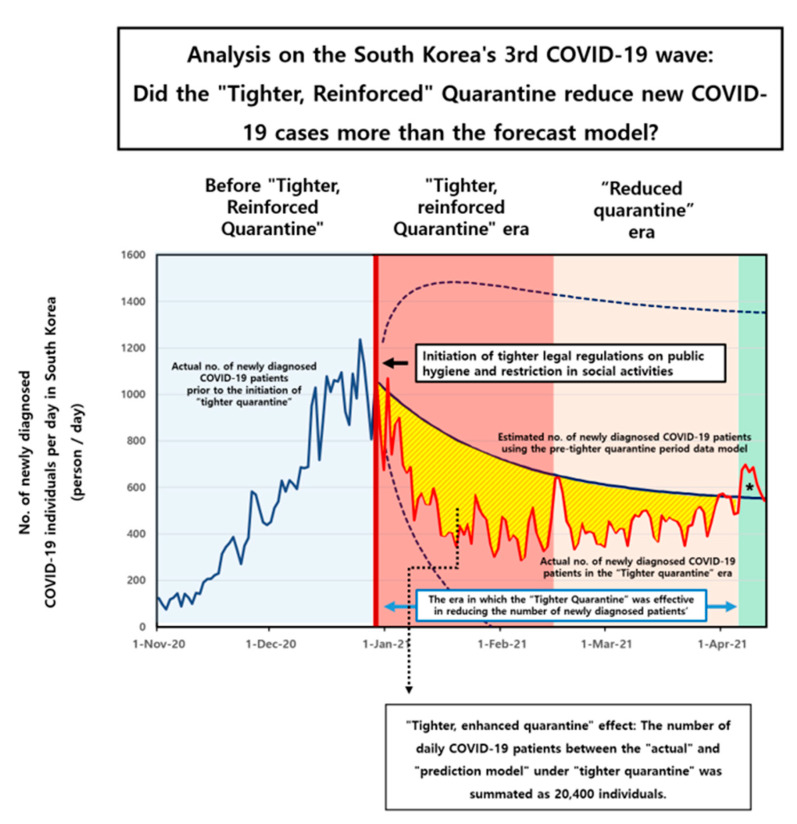
The impact of “tighter, reinforced” quarantine regulations during the 3rd wave of the coronavirus disease 2019 (COVID-19) outbreak in South Korea. The illustrated plot demonstrates the number of newly diagnosed COVID-19 individuals prior, during, and following the 3rd wave outbreak. The blue line in the light blue box area (left) indicates the actual number of newly diagnosed cases prior to the “tighter, reinforced” quarantine era. The blue bold line following the initiation of the “tighter” quarantine era indicates an estimated value of the number of newly diagnosed COVID-19 patients calculated with autoregressive integrated moving average (ARIMA) (1, 0, 0) using the pre-reinforcement era daily COVID-19 patient data. By contrast, the red bold line indicates the actual reported number of newly diagnosed COVID-19 individuals following the initiation of “tighter, reinforced” quarantine. In mid-April 2021, the measured number of newly confirmed COVID-19 cases exceeded the expected number (asterisk, light green area), suggesting that “tighter” isolation was no longer effective after three months.

The illustrated plot in Figure 2 shows the number of newly diagnosed COVID-19 individuals prior, during, and following the 3rd-wave outbreak. The expected (forecasted) number of newly diagnosed COVID-19 patients in the “tighter, reinforced quarantine period” (Figure 2, bold blue line in the ‘era of the tighter quarantine’), which was calculated with data for the daily number of newly diagnosed COVID-19 patients during the initiation of the 3rd wave until the enforcement of the tighter quarantine (Figure 2, bold blue line in the ‘prior to tighter quarantine era’) using the ARIMA (1, 0, 0) model. The blue lines with the dots represent the confidence interval for 95% of the data. On the other hand, the “actual” observed daily number of newly diagnosed COVID-19 patients during the “tighter quarantine period” (Figure 2, bold red line in the ‘tighter quarantine era’) exhibited lower numbers than the forecasted numbers in the period following the tighter quarantine regulation.

The quarantine laws were described as “tighter and strengthened,” and they were in effect for around seven weeks in a row, beginning on 24 December 2020 and ending on 17 February 2021. (Figure 2, red colored filled area). After this time, the government of South Korea loosened the restrictions placed on the quarantine region (Figure 2, area filled in a light orange color).

The prediction model’s estimate of the number of COVID-19 patients who received a new diagnosis was much higher than the actual observed number of patients who received a new diagnosis. However, the actual number of COVID-19 patients surpassed the number that was forecasted in the prediction model around four months after the beginning of the “tighter, reinforced quarantine” in April 2021 (asterisk, Figure 2).

In the approximately three-month period when the “tighter quarantine” was in force, in which the actual daily number of COVID-19 patients was less than the forecasted number, the difference in the daily number of COVID-19 patients between the “actual” and “forecast model” under “stricter quarantine” was summated as 20,400 individuals. Therefore, in our analysis, it can be anticipated that there would be an overall reduction in the number of newly diagnosed COVID-19 cases of 20,400 individuals (Figure 2, area colored in yellow), in a period of three months immediately following a tighter quarantine regulation.

Moreover, according to our results, a stricter and more strengthened quarantine had a substantial effect in the beginning but gradually had less and less of an effect as time went on. It was determined that the impact of “tighter” quarantine was no longer effective after more than three months had passed because the actual observed number exceeded the estimated number of newly diagnosed COVID-19 cases (Figure 2, asterisk, light green colored area). This suggested that after more than three months, the more strict and enhanced quarantine was no longer effective.

## 4. Discussion

During the worldwide pandemic of COVID-19, there were three waves of the outbreak in South Korea in 2020 [8] (Figure 1). The initial wave occurred in the city of Daegu and the Gyeongbuk province [16]. Since 19 February 2020, clusters of COVID-19 patients who attended religious ceremonies and activities of a particular religion were identified, resulting in about 8000 cases during the first outbreak [10]. On 23 February 2020, the national crisis awareness level was increased to its maximum status, which was the highest possible status issued by the South Korean government regarding the COVID-19 outbreak [16].

The first outbreak in the Daegu metropolitan city area was initiated by the 31st COVID-19 patient in South Korea; an epidemiologic study have revealed that patient number 31 had attended an indoor service of religious worship while having COVID-19 symptoms on 9 February 2020, as well as on 16 February 2020 [9]. Upon a thorough epidemiological investigation, it was identified that this particular religious group and their religious activities were very much more prone to the extremely rapid spreading of the COVID-19 [9]. The religious ceremony of this particular religion was held in an indoor environment most of the time. COVID-19 may spread at a very high rate, especially when numerous individuals are crowded in a limited indoor space for many hours, as was observed in an outbreak similar to this among employees in a telephone call center in South Korea [17]. During the worship ceremony, the members of this particular religion were seated in rows of seats containing over a hundred members, side by side, physically very close to one another in an indoor facility during the service of worship. Moreover, the loud singing of religious hymns and performing vocal prayers on a group level may have facilitated even more the spreading of COVID-19. As a result, the South Korean government issued a law for all religious ceremonies and activities in all religions in South Korea, effective 10 July 2020, that all members attending services should be at least one meter apart from each other, and prohibiting the performance of hymns and other vocal activities, as well as restricting group food consumption or any other culinary activities [9]. In addition, because of the secrecy with which this particular religious organization arranged their worship ceremonies, the specific identity of those who participated was often quite limited. Consequently, the rapid epidemiological action needed to stop the spread of COVID-19 during the initial epidemic was very challenging [9].

Owing to its initiation by the specific religious activity of this particular religion, the attending members contributed enormously in transmitting COVID-19 to a community level, which led to a chain reaction of COVID-19 transmission in a group of residential areas and healthcare facilities in the community [18]. In an attempt to control the massive epidemic of COVID-19 during the first outbreak, the local community members responded by voluntarily minimizing their social gathering, and the South Korean government and City of Daegu established a task force to screen and treat the infected individuals, as well as to monitor the exposed individuals based on an epidemiologic investigation. The high-intensity social distancing restriction was issued by the South Korean government beginning on the 29 February 2020, and attenuated on 30 April 2020 [18,19]. Eventually, the first outbreak was limited to the Daegu city and Gyeongbuk province [18].

The second wave started around 13 August 2020, and lasted to around 18 September 2020 [20]. To briefly elaborate on the epidemiologic background of the second wave of COVID-19 in South Korea, the level of social distancing was lowered from 6 May 2020 in response to the controlled number of COVID-19 individuals [20]. Following an outdoor rally which took place in a specific area in Seoul City on 15 August 2020, and in another religious facility in the City of Seoul, there were more than 100 confirmed cases every day, and 441 cases on 27 August 2020, mainly in the Seoul metropolitan city [10].

Owing to the fact that both the first and second waves of the disease sprang from social events or religious meetings held in certain locations, it was easy to implement quarantine procedures and keep an eye on those who were susceptible. During the first and second waves, the quarantine was mostly enforced in the region where the severity of the epidemic was greatest; it was not carried out on a countrywide scale [10]. Therefore, there has been a gradual decrease in the number of COVID-19 patients, leading to a temporary lull, which encouraged the South Korean government to lower the amount of time that people were required to remain in quarantine.

Nonetheless, the number of confirmed cases skyrocketed in the middle of December 2020, topping 600 on 4 December and 1200 on 25 December, and this epidemic was designated as the third wave of COVID-19 in South Korea [8]. In contrast to the first and second waves, the third wave featured distinct, new characteristics. The first and second waves came from a particular social or religious group; the third wave’s epidemiology was characterized by sporadic COVID-19 infections that occurred concurrently on a nationwide scale [14].

On 24 December, the South Korean government was compelled to implement a stricter degree of quarantine laws and regulations on a national scale [8]. On 11 January 2021, there were fewer than 600 newly identified cases of COVID-19 as a result of the strengthening of quarantine procedures [8]. This “tighter, strengthened” quarantine order was imposed by the South Korean government on a nationwide scale until 14 February 2021, totaling seven continuous weeks of strict quarantine laws [8]. However, the drop in newly diagnosed COVID-19 cases did not continue through to the end of February 2021, as between 400 and 600 newly diagnosed cases per day have been recorded since then [8].

Even while the number of COVID-19 cases decreased immediately after the completion of “tighter, enhanced” quarantine, this trend did not sustain until the number of new cases per day reached 400 to 600. However, even when the “tighter” quarantine was eased in March 2021, the number of newly identified COVID-19 cases did not increase and remained below the number anticipated by our analysis. Therefore, we determined that the “tighter quarantine” was effective from the beginning of January 2021 until the end of April 2021, i.e., four months. In addition, the effects of “tighter” confinement persisted for almost two months, despite the easing of quarantine laws.

During the period of the “tighter, reinforced” quarantine, everyone was required to wear face masks regardless of whether they were indoors or outdoors, gatherings of five people or more were not allowed, and an intervention was implemented to prohibit the opening of commercial establishments after a certain time had passed. In addition, gatherings of a religious, educational, or sporting nature with an anticipated attendance of more than one hundred people were prohibited by law [8]. Despite the attempts of the South Korean government to stop the spread of the COVID-19 sickness by instituting quarantine restrictions, the illness continued to be a problem in South Korea.

Supported by our analyzed results, elevating the degree of strictness of quarantine by restricting various social activities and public gatherings was shown to be effective in decreasing the number of newly diagnosed patients when the regulations were activated in the early phase during the massive outbreak, especially when the population had yet to develop immunity to the disease. Thus, in the period in which the population is susceptible to a contagious viral disease, a stricter quarantine policy should be raised to control the rapidly rising number of daily newly diagnosed cases. Nevertheless, as our results indicate, although this tighter restriction may reduce the initial explosive epidemic, it should also be considered that this would not eliminate it, nor would it be effective after more than 3 months. The authors emphasize that that the third wave occurred before COVID-19-related vaccinations were made available to the general population of South Korea; at that time period in South Korea, only healthcare personnel had begun to receive vaccinations [8]. Therefore, in order to prevent the explosive outbreaks that occurred during the third-wave period, it was essential to create a more severe quarantine system at that time. This was required since the general population had not yet acquired immunity to COVID-19 [21,22].

Vaccinating each and every individual on the face of the earth is the last and most effective step that may be taken to put an end to the epidemic [22,23]. This course of action comes highly recommended by a variety of infection control experts. In the era in which the population has achieved an immunity towards this viral disease, it should be emphasized that the effectiveness of vaccination in controlling this viral epidemic could far surpass that of imposing a stricter degree of quarantine rules pertaining to social activities and personal separation. This is something that has to be emphasized very strongly. Following the administration of immunization against COVID-19 on a countrywide basis, there is a possibility that an acquired group immunity against COVID-19 will result. In an age in which the population has gained an immunity against this human-to-human transmitted viral illness, the requirement of more stringent, strengthened regulatory limits must be evaluated. It is possible that extended and strictly enforced quarantine restrictions may place a significant financial, social, and emotional strain on the general population once the universal immunization program has been completed [24,25,26]. In light of the fact that a number of research papers have demonstrated that COVID-19 infections can be avoided by receiving vaccinations at the appropriate intervals [27,28], in order to effectively combat this viral illness, it is necessary to take into account the degree to which individuals have developed an immunity to COVID-19 [24].

Despite the fact that we have utilized the ARIMA, which is a time-series model that has been used to estimate COVID-19 trends in many previous studies [13,29,30], our work has certain limitations that must be disclosed. To begin, COVID-19 is an entirely new illness that has never been seen in the past. The historical patterns of occurrence of infectious diseases are the single most important piece of evidence for predicting the frequency with which these diseases will arise in the future. When dealing with respiratory infectious diseases, it is necessary to evaluate seasonality, which requires data spanning a number of years. The problem is that there is only one year of data available for 2020; as a result, the prediction capacity and accuracy of the model may be restricted. Second, the prevalence of the disease varies from country to country based on the length of time that people are required to remain in quarantine. In addition, the severity of the quarantine may differ from one region or time period to another. For this reason, it is better to use a time-series model that forecasts future occurrences based on current occurrence data as opposed to models that need more information and make assumptions about the disease [12]. Thirdly, the late 2020s and early 2021s were a period of time during which there was not yet a broad availability of COVID-19 immunizations [14]. In future research, it would be an interesting point of comparison to examine whether community-level-acquired immunity affects the success of improved quarantine measures. This would help to answer the question, “Does enhanced quarantine work?” The third wave of the outbreak transpired prior to the arrival of the omicron strain of SARS-CoV-2 in South Korea, which emerged around September 2021 and caused an epidemic to break out in late 2021. This occurred in South Korea [31]. In light of the fact that the omicron variant had a far greater influence on the R0 value and viral spreading potential [32], it would be interesting to evaluate the impact of stricter quarantine measures in the pre-omicron period and the omicron era in future research.

During future outbreaks of human-to-human respiratory virus diseases, especially during the early phase of the epidemic, the results of our study may give a reason and need for more stringent quarantine laws. In situations where vaccines against the specific pathogen are still being developed and have not yet been distributed, and therefore when the general population has not yet developed immunity to such viruses, a short-term intervention of tightened quarantine may be required to control the exponentially increasing number of infected people. The role of the upregulated quarantine regulations may be re-established when the vaccines are fully distributed to the general public and the entire population is predicted to develop immunity to the infectious disease, necessitating a combination with other strategies to control the disease that were not analyzed in our study but may be elucidated in future research.

## 5. Conclusions

The “tighter, reinforced” quarantine policy on a national level demonstrated its effectiveness during the third wave of the COVID-19 outbreak that occurred in South Korea in late 2020 on a national scale. This outbreak occurred at a time when the COVID-19 vaccination had not yet been disseminated to the general public. It was projected that the more stringent and strengthened quarantine would lower the number of newly diagnosed cases of COVID-19 by 20,400 persons over the following three months. When vaccines against an infection are still being produced and have not yet been disseminated, a short-term quarantine may be needed to manage the exponentially expanding number of affected persons.

## Data Availability

All data relevant to the study are included in the article.
